# Allelopathic potential impact of *Senecio angulatus* L.F. on native plants

**DOI:** 10.1080/15592324.2025.2526886

**Published:** 2025-07-20

**Authors:** Sima Sohrabi, Javid Gherekhloo, Saeid Hassanpour-Bourkheili, Farshid Ghaderi-Far, Asieh Siahmarguee, Mohammad Taheri, Sadegh Atashii

**Affiliations:** a Department of Agronomy, Ferdowsi University of Mashhad and Leader of Iranian Invasive Plants Working Group, Mashhad, Iran; b Department of Agronomy, Gorgan University of Agricultural Sciences and Natural Resources, Gorgan, Iran; c Department of Horticultural Science, Gorgan University of Agricultural Science and Natural Resources, Gorgan, Iran

**Keywords:** Alien plant, allelochemicals, aqueous extracts, inhibitory impact, initial growth

## Abstract

Urban greening had a significant effect on enhancing the distribution of alien plants, which pose a threat to the native species in new areas. *Senecio angulatus* L.f. (cape ivy) is one of the naturalized species dominant in urban ecosystems in many regions. This study was conducted to evaluate the allelopathic interference of this alien species on the native plants in these habitats in Iran. The allelopathy impact of aqueous extract of stem, leaf and root of cape ivy was estimated on germination and seedling´s growth of five native plants (*Agropyron elongatum*, *Medicago sativa*, *Portulaca oleracea*, *Silybum marianum*, and *Lactuca sativa* as indicator plant). The tested species differed in their susceptibility to allelopathy of cape ivy, in which *M. sativa*, *P. oleracea*, and *L. sativa* were significantly sensitive than other species. The inhibitory effect of aqueous extracts from leaves and stems was stronger than those from belowground parts and it revealed that the presence of higher concentrations of natural substances (phenols, flavonoids and antioxidant activity) gave it its efficiency in inhibiting the early growth of native plant. Our results imply that reducing the allelopathic impact of this species during habitat restoration requires the removal of the aboveground parts, including fallen leaves. Furthermore, the information obtained helps score cape ivy risk and impact assessment in the introduced regions.

## Introduction

Allelopathy is considered an important mechanism to recognize the negative impact of alien plant species. It also enables alien plants to establish successfully in a new area by disrupting the germination and growth of native species.^1–4^ A recent survey showed that 72 percent of the 113 invasive plant families had allelopathic potential.^2^ Alien plants can inhibit the germination, growth, and development of neighboring plants by producing secondary metabolite biochemical substances.^4–6^

The “novel weapons hypothesis” suggests that invasive plant species are more likely to be allelopathic to native vegetation. The reason for this is that native vegetation has not evolved resistance to unique allelochemicals produced by the invader that are not found in the flora of the invaded area.^7,8^ The continuously increasing rate of new introduction of alien plants makes it necessary to be more informative about their potential impact. The toxicity/allelopathic impact of alien plants is considered in all assessment systems, but in most cases, no exact data is available about newly arrived species.^9,10^ The appropriate management program will be achievable by applying assessment systems which are designed to classify the alien plants based on their negative impact on managing priorities.^11^

Cape ivy (*Senecio angulatus* L.f.) is a scandent, glabrous perennial plant from the Asteraceae family which originated from South Africa (powo.science.kew.org). It was introduced to many countries as an ornamental plant in urban ecosystems^12–15^ and has escaped from Albania, Croatia, Chile and southern California, naturalized in Portugal and Spain, and invaded Italy^16–21^ (https://dryades.units.it/). In Australia, France and New Zealand it is spreading or has spread across portions of the country.^22–24^ In Australia, it is mentioned as a significant environmental weed in Victoria, South Australia, Tasmania, New South Wales, and Western Australia (https://keyserver.lucidcentral.org). It almost occupied habitats like forest margins and coastal areas. This plant has been introduced to Iran as an ornamental plant during the current decade and distributed in many parts especially in the northern parts.^25,26^ The high potential of escaping and naturalizing along with invasion potential may be related to its easy reproduction by seed and runners.^17,27,28^ Furthermore, the toxicity potential of this species may be involved as well. The impact assessment records showed a lack of data about its environmental impact despite its high escaping records^29,30^. Knowledge about its deleterious impact on germination and growth of native plants will be vital to obtain accurate output for impact and risk assessment systems.^31,32^ Furthermore, the subsequent distribution and successful establishment will be affected by its allelochemicals. The objective of this study is to discover the possible involvement of allelopathic impact of cape ivy plant parts (leaves, stems, roots and rhizomes) on germination and early growth of native plants. We hypothesized that (1) the native species differ in terms of seed germination and seedling growth under the allelopathic effects of cape ivy extract, (2) the impacts on germination and growth of native species vary depending on the plant parts, and (3) the contents of total phenols, flavonoids and antioxidants in different plant parts vary.

## Materials and methods

Laboratory experiments were conducted as factorial in a completely randomized design with three replicates to examine the effect of *S. angulatus* leaf, stem and root + rhizomes extracts (Factor A) at 0, 25, 50, 75, and 100% concentration levels (Factor B) on the germination and seedling growth of the five native plant species (Factor C, Table 1) at Gorgan University of Agricultural Sciences and Natural Resources, Gorgan, Iran. We mixed the root and rhizomes due to the lack of adequate rhizomes and the same allelochemical potential of root and rhizomes of Asteraceae species.^3^

**Table 1.** The native species used for the experiment.

Species name	Common name	Family
*Agropyron elongatum* (Host) P.Beauv.	Tall Wheatgrass	Poaceae
*Medicago sativa* L.	Alfalfa	Fabaceae
*Portulaca oleracea* L.	Purslane	Portulacaceae
*Silybum marianum* (L.) Gaertn.	milk thistle	Asteraceae
*Lactuca sativa* L.	Lettuce	Asteraceae

### Collection of plant materials

The roots, rhizomes, stems, and leaves were collected in March 2024 in Gorgan (36°84′18″ N, 54º43′34 E), Iran. Five native species of Iran were used in the study. All seeds did not need dormancy breaking (Table 1). These species are typical in urban areas, and agricultural and semi-natural grasslands in North of Iran, which are important for pollinators and grow in similar environmental conditions as Cape ivy.

### Preparation of aqueous extract

The leaf, stem, and root samples (approximately 500 grams) were stayed in the shade at a room temperature of 25°C for roughly one month. Afterward, the dried samples were ground into a fine powder with a mechanical grinder. For preparation of aqueous extracts, the ground materials from the leaf, stem, and root samples of *S. angulatus* were combined with distilled water in a 1:10 w/v ratio. To achieve various concentrations of 25, 50, 75, and 100 percent, both crude extracts (ml) were diluted with distilled water (ml) in the following ratios: 25 ml of extract to 75 ml of distilled water, 50 ml of extract to 50 ml of distilled water, 75 ml of extract to 25 ml of distilled water, and 100 ml of extract to 0 ml of distilled water, respectively (assuming 25 ml, 50 ml, 75 ml, and 100 ml extracts equivalent to 25, 50, 75, and 100 percent concentrations, respectively). The concentrations were subjected to shaking on an orbital shaker at a speed of 150 rpm for 24 hours at room temperature. Following this, the samples were filtered using filter paper, and four different concentrations (25, 50, 75, and 100%) of the aqueous extracts from each part were created. The extracts were stored in the refrigerator at a temperature of 4°C during the experiment.

### Quantification of allelochemicals

#### Total phenols

The total phenolic content was assessed using the Folin – Ciocalteu reagent method.^33^ A 1 mL sample of the extract, diluted in methanol (1 mg/mL, with three replicates for each sample), was measured, followed by the addition of 500 μL of Folin – Ciocalteu reagent and 6 mL of distilled water. This mixture was stirred for 5 minutes, after which 1.5 mL of Na2CO3 (20%) and 1.9 mL of distilled water were incorporated while stirring to ensure proper mixing. After being incubated in the dark for 2 hours, the absorbance was noted at 760 nm using a UV-30 spectrophotometer. The blank was prepared by substituting the diluted extract with methanol. Results are presented as mg of gallic acid equivalents (GAE) per mg of dry weight.

#### Total flavonoids

The total flavonoids were extracted using the same method as that for phenols, with the exception that the sample was re-suspended in 80% ethanol at a concentration of 1 mg/mL. For the quantification of flavonoids, 2 mL of ethanol was measured, to which 0.5 mL of the extract (1 mg/mL re-suspended in 80% ethanol, with three replicates) and 0.15 mL of NaNO2 (1 M in ethanol) were added. The mixture was stirred and allowed to settle for 3 minutes. After that, 0.15 mL of AlCl3 (10% in ethanol) was added, and the sample was stirred for another 3 minutes. Next, 1 mL of NaOH (1 M in distilled water) was added, along with ethanol to reach a total volume of 5 mL. The solutions were mixed again after these additions and kept in the dark for 40 minutes.^34^ The absorbance was measured at 415 nm using a UV-30 spectrophotometer. A blank was created by substituting the diluted extract with ethanol. The results are described in mg of quercetin equivalents (QE) per mg of dry weight.

#### Antioxidant activity

The antioxidant properties of the plant extracts were evaluated against DPPH using the method outlined by Katalinic et al.^35^ A solution of DPPH at a concentration of 1 × 10 − 4 M was created using methanol. For each sample in the methanolic extract, 1 mL aliquots were taken at four different concentrations, with three replicates for each sample and concentration, and were combined with 2 mL of the DPPH methanolic dilution. This mixture was kept in the dark at ambient temperature for 16 minutes, after which the absorbance was recorded at 517 nm using a UV-30 spectrophotometer (GIORGIO-BORMAC SRL, Carpi, Italy). The blank was made using the methanolic DPPH dilution.

### Allelopathic bioassay

25 test plant species seeds were arranged in 9 cm diameter Petri dishes with one piece of filter paper. Five milliliters of water extracts of *S. angulatus* leaf, stem, and root samples in diverse concentrations (25, 50, 75, and 100 percent) were used to moisten the Petri dishes. After preparing, they were placed in the incubator at random. Based on the ideal temperature for each species, they were maintained at 20 to 25 C for 14 days, with a photoperiod of 16 hours of light and 8 hours of darkness. In each replication, the number of seeds that germinated during this period was counted (germination was defined as a visible radicle with a length ≥2.00 mm). The length of the roots and stems of ten randomly chosen seedlings per treatment were also measured using a linear scale from stem base to root tip (root length), and stem base to plumular hook (shoot length).

### Statistical analysis

The response index (RI) for the measured traits is calculated using Equation 1:^36^

If *T* < C then RI = $\frac{T}{C}-1$

If *T* > C then RI = $1-\frac{C}{T}$

In which T and C are the measured values for treatments and the average of control in each species, respectively. The response index varies from −1 to + 1. Positive values indicate an increase due to the treatment, whereas negative values imply that the treatment has an inhibitory effect compared with the control. Analysis of variance was performed on the data using the SAS v.9.0 software (Supplementary tables S1 to S4). The least significant difference (LSD) method at *p* < 0.05 was carried out for comparison of means. Figures were prepared using Microsoft Excel v. 2016 and SigmaPlot v.12.5 software.

## Result

### Different species response

**Figure 1.**
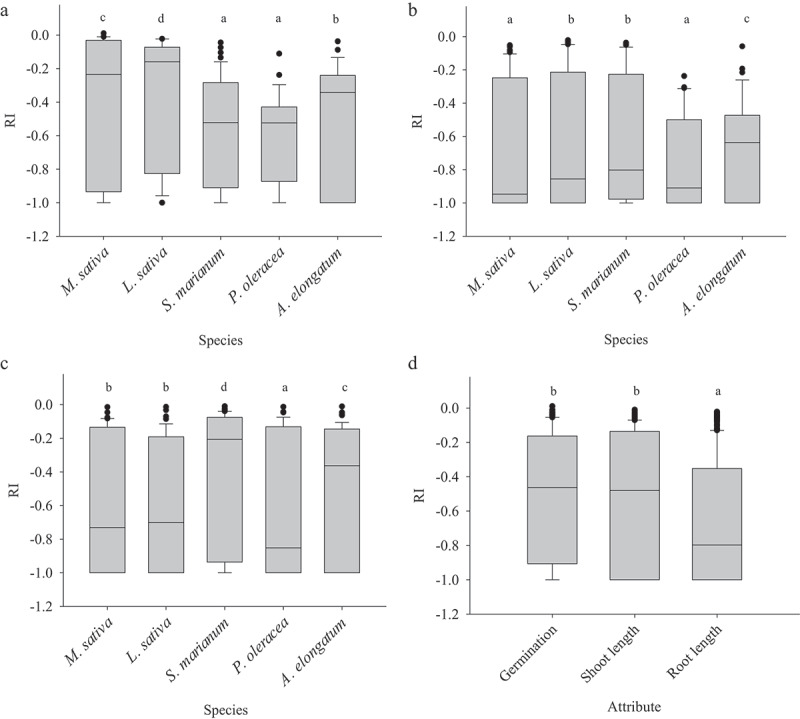
Response index for germination (a), root length (b), shoot length (c), and overall RI (d) in different species. Similar letters denote non-significance at *p* < 0.05.

The aqueous extract of *S. angulatus* caused reductions in seed germination and early growth of all tested native species. The negative impact on seed germination and early growth was expressed as negative values of response index (RI). The greatest reduction for germination was detected for *P. oleracea* (−0.523) and *S. marianum* (−0.522), and these two species showed significant differences with other species. Root growth of *M. sativa* (−0.946) and *P. oleracea* (−0.910) were more affected than the rest tested species. The lowest and highest shoot growth were discovered in *P. oleracea* (−0.852) and *S. marianum* (−0.206), respectively. In general, root growth was more affected than germination and shoot growth due to having a lower median (−0.797) (Figure 1d).

**Figure:**
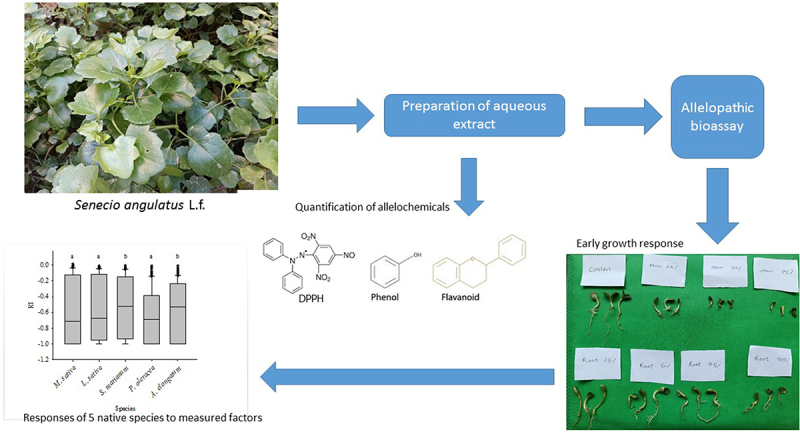
The overall procedure of examining the allelopathic impact of *Senecio angulatus*

**Figure 2.**
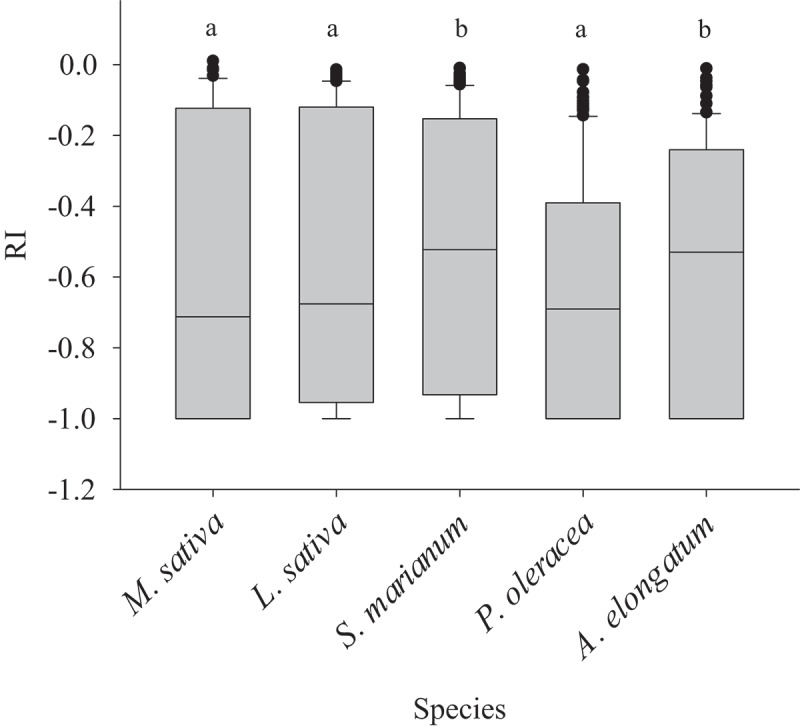
Responses of various species to all measured factors. Similar letters denote non-significance at *p* < 0.05.

Among five native species *M. sativa*, *P. oleracea*, and *L. sativa* were most sensitive to allelopathic inhibition for all measured factors (Figure 2). While *A. elongatum* and *S. marianum* showed the lowest sensitivity.

### Germination response

**Figure 3.**
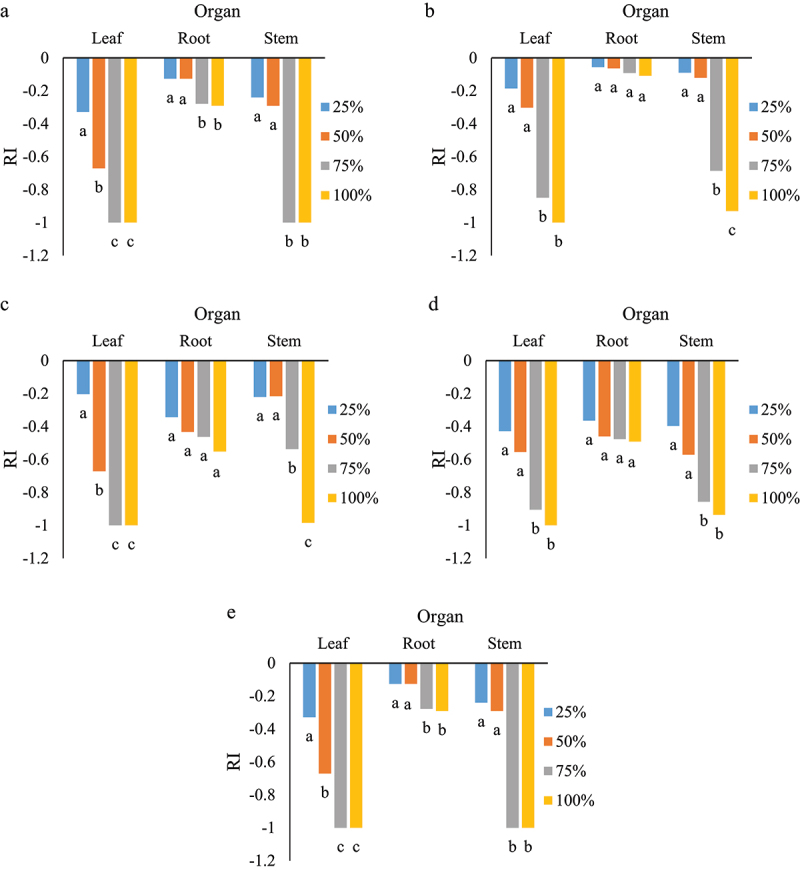
Response index of germination as affected by various concentrations obtained from leaves, roots and stems on *M. sativa* (a), *L. sativa* (b), *S. marianum* (c), *P. oleracea* (d), and *A. elongatum* (e). Similar letters in each organ denote non-significance at *p* < 0.05.

Response index of germination as affected by various concentrations of leaves, roots and stems of *S. angulatus*. The highest reduction was observed in the aqueous extract of leaves and stems of *S. angulatus* (Figure 3). Different responses were detected in different concentrations of aqueous extract. For *M. sativa*, there was a significant difference between 25, 50 and 75% concentrations in the aqueous extract of leaves and the response index was similar at 75 and 100%. No significant differences were observed among the different root extract concentrations of cape ivy (Figure 3a). *Lactuca sativa*, as an indicator plant, was shown to have the highest germination reduction at concentrations of 75 and 100% of leaf aqueous extract (Figure 3b). This trend was similar for *S. marianum* and *P. oleracea* (Figure 3c,d). In *A. elongatum*, germination reached a minimum in 75 and 100% concentrations of leaf and stem extracts (Figure 3e). A drastic reduction (RI value = −0.7) of germination was seen at 50% in *M*. *sativa*, *S. marianum* and *A. elongatum*.

### Early growth response

**Figure 4.**
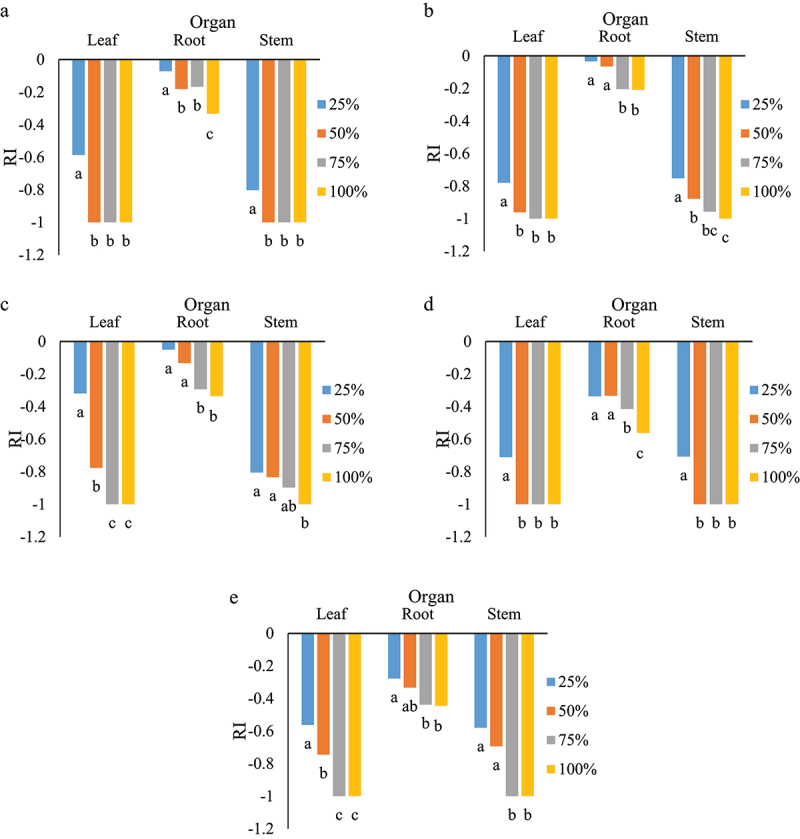
Response index of root length as affected by various concentrations obtained from leaves, roots and stems on *M. sativa* (a), *L. sativa* (b), *S. marianum* (c), *P. oleracea* (d), and *A. elongatum* (e). Similar letters in each organ denote non-significance at *p* < 0.05.

**Figure 5.**
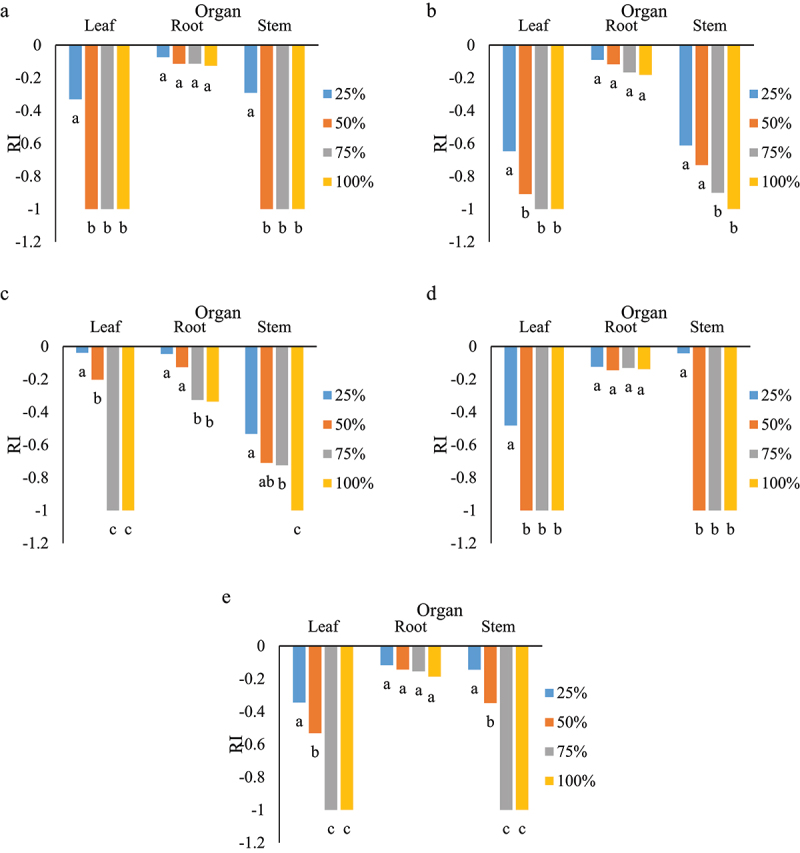
Response index of shoot length as affected by various concentrations obtained from leaves, roots and stems on *M. sativa* (a), *L. sativa* (b), *S. marianum* (c), *P. oleracea* (d), and *A. elongatum* (e). Similar letters in each organ denote non-significance at *p* < 0.05.

The early growth of all examined species was reduced with increasing concentrations of aqueous extract of *S. angulatus* (Figure 4). At 25% of aqueous extract of leaves, the lowest response index was detected in *L. sativa* and *P. oleracea*. At 25% of the aqueous extract of stems, the RI values for *M. sativa* and *S. marianum* were about −0.8 (Figure 4). The root growth of *A. elongatum* was less affected in comparison with the other species (Figure 4e). The significant difference in early growth response can be related to the greater response of 50% concentration. In most species apart from *A. elongatum* and *S. marianum*, there was no significant difference between 50, 75 and 100% concentrations especially, in leaf extract of cape ivy (supplementary figure s1). Shoot growth was affected by different organs and concentrations of *S. angulatus* (Figure 5). At 25% of the aqueous extract of leaves and stems, *L. sativa* showed the highest reduction of shoot growth compared to the other species (Figure 5b and supplementary figure S2). Our results also revealed the negative impact of root extract of *S. angulatus* on the shoot growth of *P. oleracea* (Figure 5d and supplementary figure S3).

### *The allelochemicals of* S. angulatus

**Figure 6.**
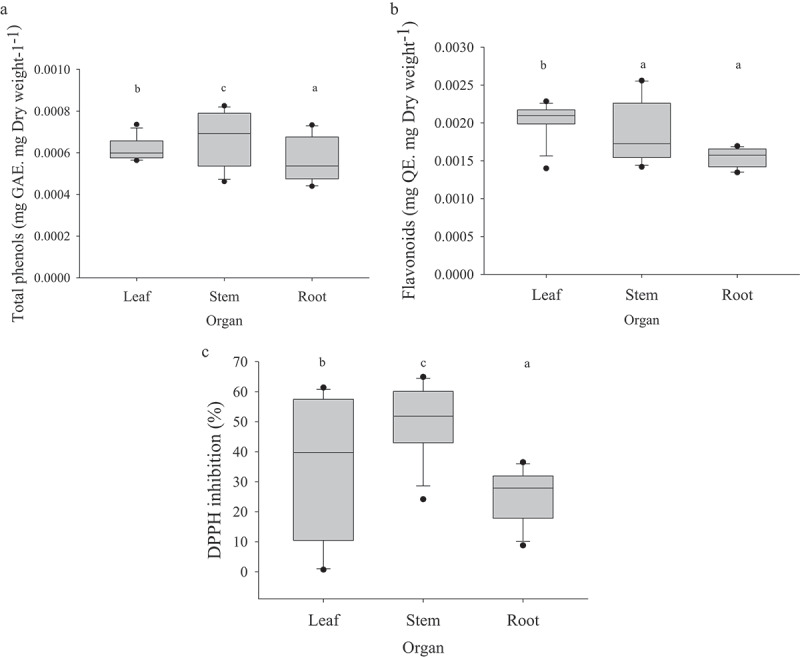
Total phenols (a), flavonoids (b) and DPPH inhibition in different plant organs. Similar letters in each organ denote non-significance at *p* < 0.05.

The total phenols, flavonoids and DPPH varied depending on the different organs of *S. angulatus* (Figure 6). The highest and lowest phenols were detected in the stems and roots of *S. angulatus* with 0.00069 and 0.00053 mg GAE. mg dry weight^−1^, respectively. The highest levels of phenols, flavonoids and DPPH were observed in the stem, leaf (0.002 mg QE. mg dry weight^−1^), and stem (51.88 %) of *S. angulatus*, respectively. There was a significant difference between the allelochemicals of organs of S. *angulatu*s (Table 2).

**Table 2.** Comparison of means for the content of some allelochemicals in different plant organs under various concentrations.

Organ	Concentration	Total phenols (mg/mg dw)	DPPH (%)	Flavonoids (mg/mg dw)
Stem	25%	0.0004906667^c^	61.084024^a^	0.001480700^c^
	50%	0.0006279000^b^	37.558578^b^	0.001590133^c^
	75%	0.0007996333^a^	58.522514^a^	0.001873767^b^
	100%	0.0007605333^a^	45.411628^b^	0.002492400^a^
Leaves	25%	0.0005696333^b^	2.742265^c^	0.001980967^ab^
	50%	0.0005773000^b^	25.458814^b^	0.001846967^b^
	75%	0.0006838667^a^	57.747321^a^	0.002186433^a^
	100%	0.0006440000^a^	56.196933^a^	0.002155167^a^
Root	25%	0.0004531000^b^	30.042568^a^	0.001364567^b^
	50%	0.0004860667^b^	31.222211^a^	0.001529833^a^
	75%	0.0006340333^a^	12.786080^b^	0.001634800^a^
	100%	0.0006831000^a^	28.289956^a^	0.001659367^a^

Similar letters in each column and each organ denote non-significance at *p* < 0.05.

## Discussion

Many studies report the negative effect of water extracts from alien plant species on the germination and growth of native plants.^5,37–39^ Our results revealed that the tested species differ in susceptibility to *S. angulatus* allelopathy. The elevated inhibition of seeds treated at lower concentrations of aqueous shoot extract of *S. angulatus* possibly indicated that this species was more sensitive. Shoot growth of *M. sativa* was more sensitive to allelopathy of *S. angulatus*. There are reports that legume species are more sensitive to allelopathy impact.^3,5^ The most resistant species in our experiment were *L. sativa* at the germination stage and *S. marianum* and *A. elongatum* at the early growth stage. The obtained results suggest that the above-mentioned species can be prioritized when invaded areas are restored by cape ivy. The reduction in the vegetation coverage of the native species may be related to decreased germination and growth performance due to allelopathy interference of invaded species.^40,41^

Germination is mentioned as a sensitive stage of plant growth that is highly affected by allelochemicals. The disruption of metabolism and cell functions by allelochemicals may cause the inhibition of germination.^42,43^ The expansion of invasive plant species in the introduced areas will be facilitated by inhibiting the germination and growth of native plants.^44^ Two annual invasive plants *Ambrosia artemisiifolia* L. and *Ambrosia trifida* L. had significant autotoxicity.^45^ This can inhibit their seed germination, prevent seedling competition and sustain the expansion of their population away from the parent plants. Besides, the inhibiting of chlorophyll synthesis in target plants was reported as one of the key mechanisms of *Artemisia argyi* H. Lev. & Vaniot to inhibit the germination and growth of other plants.^46^ In general, allelopathy is introduced as a mechanism for the rapid spread of some naturalized and invasive ornamental plants such as *Mirabilis jalapa* L,^47^ *Lantana camara* L,^40^ *Amorpha fruticosa* L. and *Asclepias syriaca* L.^48^

The allelopathic effects of aqueous extracts were dependent on concentration. The exerted significant inhibitions at 50% to 100% were more highlighted in many allelopathic studies.^38,49,50^ The inhibitory response was also dependent on the organ, and the allelopathic effect of the aboveground organ was reported to be stronger than that of the underground organ in many studies.^3,39,47,51,52^ Effah and Clavijo McCormick^7^ showed that a plant’s vulnerability to allelochemicals depends both on the specific organ exposed and on the concentration of those compounds. This is particularly the case when the plant hasn’t co‑evolved with the allelochemical source.

It is suggested that the higher content of phenol and flavonoids in leaf and stem extract was possibly attributed to the inhibitory impact of cape ivy on germination and early growth of the native plants. Phenolic compounds differ in plant tissues, including leaves, flowers, stem, and roots.^40,53^ In some Asteraceae species, the inhibitory effect of water extracts of leaves and flowers was stronger than those of rhizomes and roots.^3,52^ Phenolic and flavonoid compounds are reported previously as main groups of secondary metabolites in *Senecio* species.^54,55^ The phytochemical screening of the extracts of *Senecio angulatus* revealed a higher amount of cynarin and chlorogenic acid as phenolic compounds.^56^ The tribe Senecioneae is known to produce pyrrolizidine alkaloids.^57,58^ Phenolic compounds with hydroxyl in their structure in cape ivy may significantly contribute to antioxidant activity.^56^ Some phenolic acids are introduced as an important compound in *Ligularia sagitta* Maxim. as an invasive plant.^4^ The results also showed that root extract had antioxidant activity (20%), while 80% antioxidant activity was determined in the shoot extract, especially stem extract (50%). Antioxidants are secondary metabolites as total phenol that can protect cells from damage caused by free radicals and can react with superoxide anions and lipid peroxyl radicals and so inhibit lipid peroxidation.^6^

Cape ivy as an invasive species in many areas poses a potentially major threat to biodiversity because it outcompetes native vegetation. It has been described as the biggest threat to natural ecosystems and is capable of destroying upper-story vegetation.^14,17,59^ It is recommended to regenerate native plants on nearby sites to minimize their negative impact.^60^ The result of this work is the first evidence of the allelopathy impact of cape ivy and the output will help score its risk and impact assessment. As a result, this species can be ranked a medium impact based on EICAT (Environmental Impact Classification for Alien Taxa). There is a strong need to study the allelopathic effects of cape ivy in the soil to assess the interactions of particular conditions in the soil and other native plant response.

## Conclusion

This research contributes to a substantial collection of studies indicating that allelopathy serves as a mechanism for the dominance of invasive plants, which can modify plant communities by suppressing the growth and development of native species. The results of the study showed that allelo-chemicals were mainly present in the aqueous extracts of stems and leaves of *S. angulatus*. In accordance with this study, subsequent research will be conducted to isolate and purify the aqueous and ethyl acetate extracts in order to identify the allelochemicals present in this plant and to further explore the specific mechanisms of allelopathy. Therefore, future control and management initiatives, along with fundamental ecological and coexistence research, should prioritize a broader range of native species, particularly in relation to soil conditions.^47^

## Supplementary Material

Supplemental Material
