# Supplementary material for: Allelopathic potential impact of Senecio angulatus L.F. on native plants
Source: Plant Signal Behav. 2025 Jul 20;20(1):2526886. doi: 10.1080/15592324.2025.2526886 (PMC12283011; doi:10.1080/15592324.2025.2526886)
Supplement: Supplemental Material [file KPSB_A_2526886_SM9160.docx]

Supplementary figures:


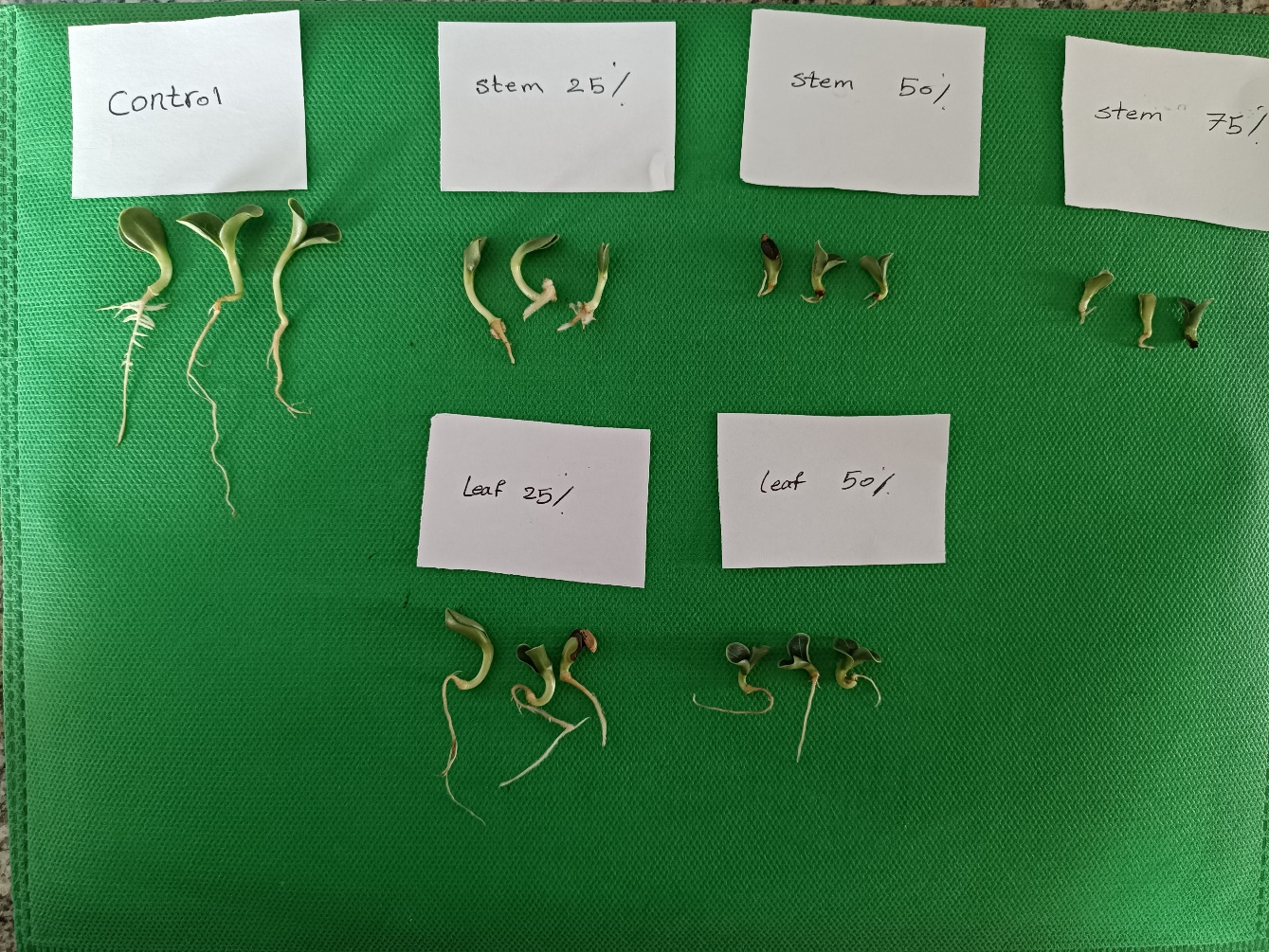


Figure1: The growth of *Silybum marianum* seedlings under aqueous extract of cape ivy. The seedlings in root extract are not shown. Seeds of *Silybum marinum* had no germination in 75 and 100% of leaf extract and 100% of stem extract.


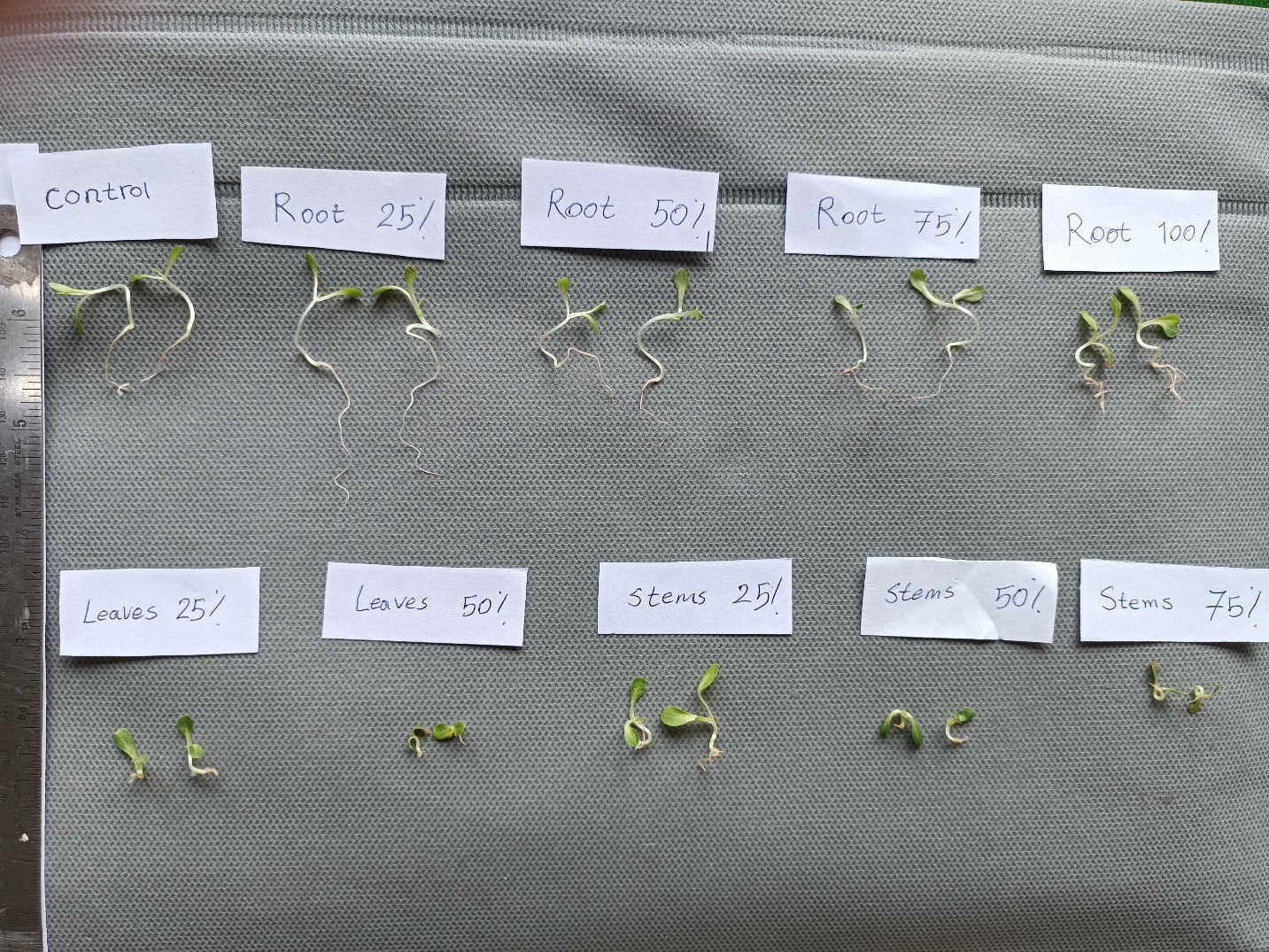


Figure 2: The growth of *Lactuca sative* seedling under aqueous extract of cape ivy. Seeds of *L. sative* had no germination in 75 and 100% of leaf extract and 100% of stem extract.

| 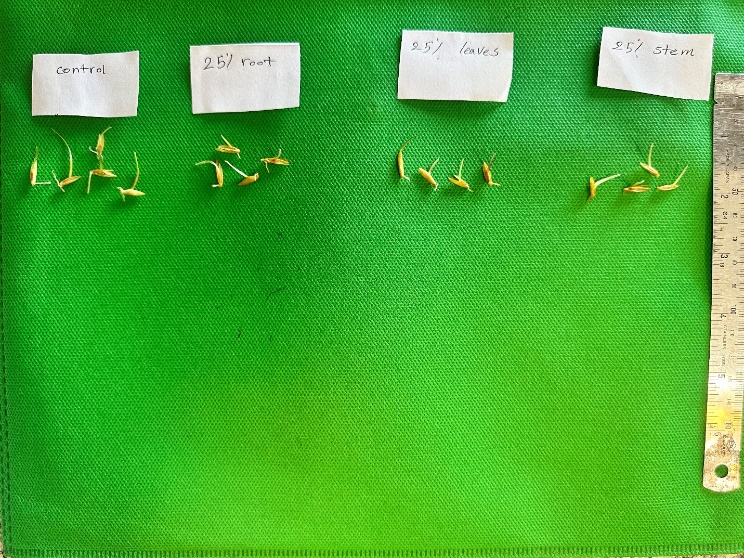 | 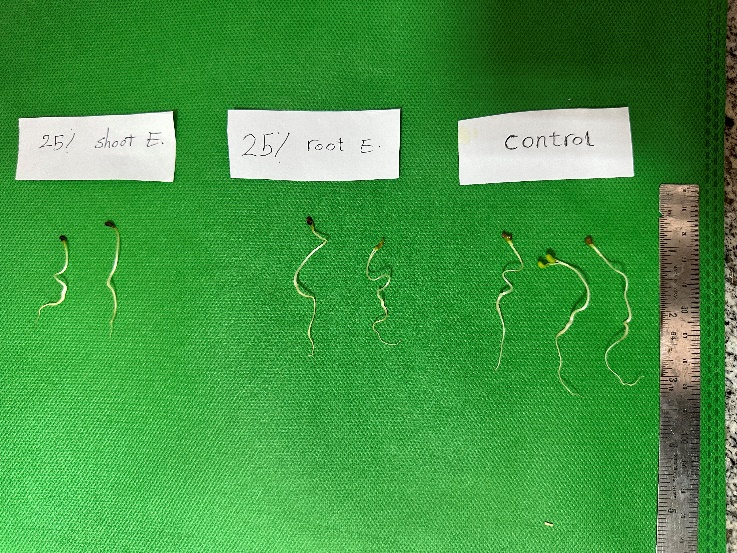 |
| --- | --- |


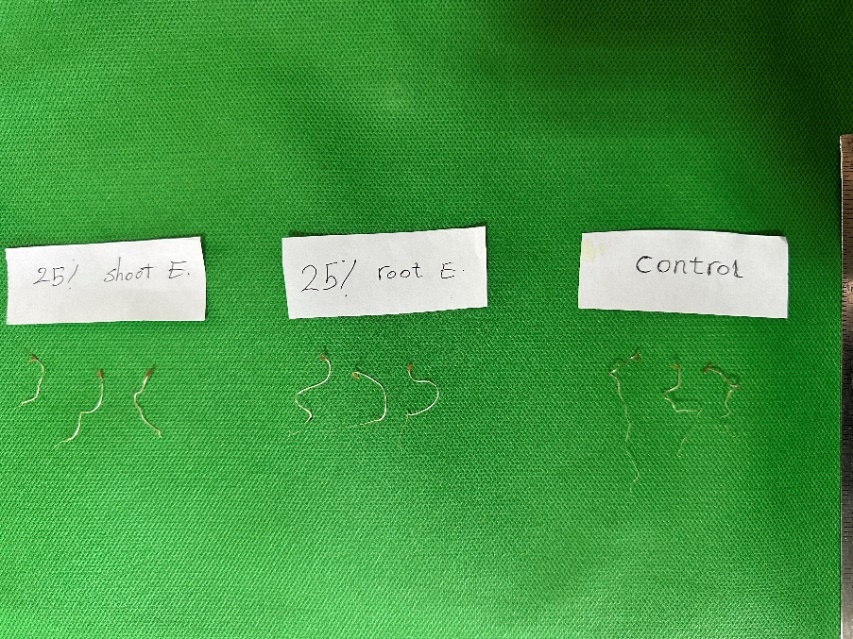


Figure 3: The growth of *Agropyron elongatum*, *Medicago sativa* and *Portulaca oleracea* under different concentration of aqueous extract of cape ivy. All concentration is not shown.

Supplementary material

Table S1. Analysis of variance for the response index of germination percentage for the studied species as affected by extracts obtained from various organs and concentrations of cape ivy.

| S.O.V | DF | Mean squares | | | | |
| --- | --- | --- | --- | --- | --- | --- |
|  |  | *Medicago sativa* | *Lactuca sativa* | *Silybum marianum* | *Portulaca oleracea* | *Agropyron elongatum* |
| Organ | 2 | 1.841 ^**^ | 1.099 ^**^ | 0.340 ^**^ | 0.358 ^**^ | 1.313 ^**^ |
| Concentration | 3 | 0.959 ^**^ | 0.943 ^**^ | 0.556 ^**^ | 0.439 ^**^ | 0.895 ^**^ |
| Organ × Concentration | 6 | 0.253 ^**^ | 0.199 ^**^ | 0.283 ^**^ | 0.062 ^**^ | 0.134 ^**^ |
| Error | 36 | 0.003 | 0.007 | 0.017 | 0.016 | 0.005 |
| Total | 47 | --- | --- | --- | --- | --- |
| CV (%) | --- | -13.87 | -23.43 | -23.62 | -20.55 | -13.52 |

** significant at p<0.01.

Table S2. Analysis of variance for the response index of root length for the studied species as affected by extracts obtained from various organs and concentrations of cape ivy.

| S.O.V | DF | Mean squares | | | | |
| --- | --- | --- | --- | --- | --- | --- |
|  |  | *Medicago sativa* | *Lactuca sativa* | *Silybum marianum* | *Portulaca oleracea* | *Agropyron elongatum* |
| Organ | 2 | 2.893 ^**^ | 3.316 ^**^ | 2.129 ^**^ | 1.414 ^**^ | 1.076 ^**^ |
| Concentration | 3 | 0.203 ^**^ | 0.115 ^**^ | 0.343 ^**^ | 0.167 ^**^ | 0.345 ^**^ |
| Organ × Concentration | 6 | 0.026 ^**^ | 0.004 ^**^ | 0.084 ^**^ | 0.024 ^**^ | 0.024 ^**^ |
| Error | 36 | 0.002 | 0.001 | 0.009 | 0.003 | 0.006 |
| Total | 47 | --- | --- | --- | --- | --- |
| CV (%) | --- | -7.43 | -5.64 | -15.78 | -7.12 | -12.24 |

** significant at p<0.01.

Table S3. Analysis of variance for the response index of shoot length for the studied species as affected by extracts obtained from various organs and concentrations of cape ivy.

| S.O.V | DF | Mean squares | | | | |
| --- | --- | --- | --- | --- | --- | --- |
|  |  | *Medicago sativa* | *Lactuca sativa* | *Silybum marianum* | *Portulaca oleracea* | *Agropyron elongatum* |
| Organ | 2 | 2.714 ^**^ | 2.534 ^**^ | 0.535 ^**^ | 2.518 ^**^ | 1.436 ^**^ |
| Concentration | 3 | 0.683 ^**^ | 0.218 ^**^ | 1.651 ^**^ | 0.741 ^**^ | 0.837 ^**^ |
| Organ × Concentration | 6 | 0.135 ^**^ | 0.020 ^**^ | 1.173 ^**^ | 0.223 ^**^ | 0.198 ^**^ |
| Error | 36 | 0.002 | 0.004 | 0.006 | 0.003 | 0.004 |
| Total | 47 | --- | --- | --- | --- | --- |
| CV (%) | --- | -8.42 | -11.11 | -18.88 | -10.28 | -13.61 |

** significant at p<0.01.

Table S4. Analysis of variance for the content of some allelochemicals in the extracts obtained from various organs and concentrations of cape ivy.

| S.O.V | DF | Mean squares | | |
| --- | --- | --- | --- | --- |
|  |  | Flavonoids | Phenols | DPPH |
| Organ | 2 | 0.000000752 ^**^ | 0.000000033 ^**^ | 1910.44 ^**^ |
| Concentration | 3 | 0.000000472 ^**^ | 0.000000089 ^**^ | 418.40 ^**^ |
| Organ × Concentration | 6 | 0.000000137 ^**^ | 0.000000008 ^**^ | 1135.62 ^**^ |
| Error | 36 | 0.000000015 | 0.000000001 |  |
| Total | 47 | --- | --- | --- |
| CV (%) | --- | 6.81 | 5.83 | 14.09 |

** significant at p<0.01.
